# Seed dormancy, climate changes, desertification and soil use transformation threaten the Mediterranean endemic monospecific plant *Petagnaea gussonei*

**DOI:** 10.1038/s41598-024-58948-1

**Published:** 2024-04-08

**Authors:** Giuseppe Bonanno, Vincenzo Veneziano

**Affiliations:** Department of Biological, Geological and Environmental Sciences, University of Catania, Via Antonino Longo 19, 95125 Catania, Italy

**Keywords:** *Petagnaea gussonei*, Mediterranean endemism, Seed biology, Climate trends, Environmentally sensitive areas, CORINE Land Cover, Plant sciences, Biodiversity, Conservation biology

## Abstract

This study investigated the germination capacity (endogenous factor) of *Petagnaea gussonei* (Spreng.) Rauschert, an endemic monospecific plant considered as a relict species of the ancient Mediterranean Tertiary flora. This investigation focused also on the temporal trends of soil-use, climate and desertification (exogenous factors) across the natural range of *P. gussonei*. The final germination percentage showed low values between 14 and 32%, the latter obtained with GA_3_ and agar at 10 °C. The rising temperatures in the study area will further increase the dormancy of *P. gussonei*, whose germination capacity was lower and slower at temperatures higher than 10 °C. A further limiting factor of *P. gussonei* is its dormancy, which seems to be morpho-physiological. Regarding climate trends, in the period 1931–2020, the average temperature increased by 0.5 °C, from 15.4 to 15.9 °C, in line with the projected climate changes throughout the twenty-first century across the Mediterranean region. The average annual rainfall showed a relatively constant value of c. 900 mm, but extreme events grew considerably in the period 1991–2020. Similarly, the land affected by desertification expanded in an alarming way, by increasing from 21.2% in 2000 to 47.3% in 2020. Soil-use changes created also a complex impacting mosaic where c. 40% are agricultural areas. The effective conservation of *P. gussonei* should be multilateral by relying on germplasm banks, improving landscape connectivity and vegetation cover, and promoting climate policies.

## Introduction

Human activities are drastically altering the planet’s biosphere, leading to an unprecedented biodiversity crisis^1,2^. With nearly three-quarters of Earth’s surface altered by human activities^3^, the degradation of ecosystems and natural habitats is continuing at an alarming pace, despite decades of effort to achieve all targets aimed at halting biodiversity loss^4^. The Mediterranean region, in particular, is a world hotspot of plant biodiversity that hosts 10% of all known vascular plants, amounting to c. 25,000 species of which 13,000 are endemic taxa^5–7^. Endemic plants are of great interest for a variety of reasons that encompass inherent geographic limitation, usually threatened conservation status, and important ecological roles such as specialist pollinators and/or dispersers^8^. The Mediterranean region is thus a key area for the conservation of global plant biodiversity. However, effective measures of species conservation require a multivariate approach that considers factors acting both intrinsically (endogenously) and extrinsically (exogenously) on biodiversity. Seed dormancy, for example, is a complicated feature of plants that reflects how a species adapted to its habitat conditions^9,10^, consequently, germination behavior is very important for the conservation of plant species^11,12^. Climate change is accelerating biodiversity loss at an alarming rate^3^, and is likely to intensify over the next few decades unless substantive mitigation efforts are implemented^13^. The Mediterranean region has been especially identified as highly vulnerable to desertification^14^, triggered by deep soil-use changes^15^, and high sensitivity to climate changes^13,16^. The resulting habitat fragmentation reduces landscape connectivity, and increases extinction risk^17^.

This study investigated some important endogenous and exogenous factors that can influence the conservation strategy of any plant species: seed dormancy, climate trends, desertification and soil-use changes. This study focused in particular on *Petagnaea gussonei* (Spreng.) Rauschert, a Mediterranean endemic monospecific plant (fam. *Apiaceae*, subfam. *Saniculoideae*, syn. *Petagnia saniculifolia* Guss., *Sison gussonii* Spreng., *Sison gussonianum* Balb. ex DC., *Petagnaea saniculaefolia* (Spreng.) Caruel). Specifically, this species is considered as a palaeoendemic relict of the Sicilian Tertiary flora^18^, narrowly distributed across the north-eastern mountains of Sicily (Italy). *Petagnaea gussonei* is categorized as Endangered (EN) according to the IUCN Red List Criteria^19,20^. It is listed in Appendix I of the Bern Convention^21^, and in Annexes II and IV of the Habitats Directive 92/43/EEC^22^. *Petagnaea gussonei* has been also included among the “Top 50 Mediterranean Island Plants”^23^. Although *P. gussonei* is protected by local and national laws and conventions*,* measures for the conservation of this endangered plant species are only slowly being introduced. Moreover, *P. gussonei* has been poorly investigated with respect to its germination behavior^24^. Knowledge of seed dormancy and optimal propagation techniques are indeed crucial for implementing conservation programs to counteract the risk of species extinction, and for successful restoration and reintroduction projects. Similarly, despite the endangered conservation status of this plant species, no study examined the climate and soil-use temporal changes occurred within the distributional area of *P. gussonei*. This endemic species should be a priority for conservation biology in Sicily, and effective protection strategies and management actions should ensure the persistence of *P. gussonei* under current and future climate and environmental changes. This study specifically aimed to shed light both on the seed dormancy of *P. gussonei*, and on the multi-temporal trends of climate, desertification and soil-use that affect the natural range size of *P. gussonei*.

## Materials and methods

### Study area

**Figure 1:**
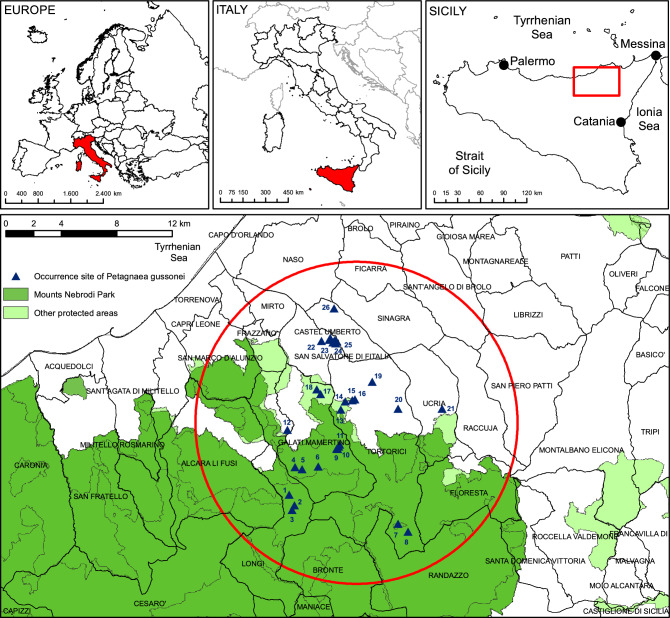
Study area that shows the natural range of *Petagnaea gussonei* (red circle).

This study was conducted in north-eastern Sicily (Italy), within the Nebrodi Mountains (province of Messina, Fig. 1). The Nebrodi are geomorphologically complex, and occupy the central sector of the orographic chain along northern Sicily, which is the natural extension of the Italian Apennine Mountains. The Nebrodi stretch for c. 70 km, and their highest peak is Mount Soro (1847 m asl), surrounded by a vast beechwood. Distinctive features of the Nebrodi landscape are the so called *fiumare*, namely short, wide gravel-bed streams characterized by steep slopes, a large mouth, a seasonally variable flow and a torrential regime with catastrophic transport of solid material following heavy rainfall. In 1993, the Sicilian regional government created the Nebrodi Park, the largest protected area in Sicily (c. 86,000 ha). From a geological point of view, the area is part of a collisional system, developed since the Late Cretaceous, as the result of the convergence between the European and African-Adriatic plates^25,26^. The climate is Mediterranean, with a long and dry summer. The annual mean temperature is c. 15 °C (with a minimum of − 5 °C in winter and a maximum of 35 °C in summer); the annual rainfall ranges from 600 to 1300 mm. From a bioclimatic point of view ^27^, the territory investigated lies in the oceanic pluviseasonal Mediterranean belt, with meso-supramediterranean thermotype and subhumid-humid ombrotype.

### Biology and ecology of *Petagnaea gussonei*

**Figure 2:**
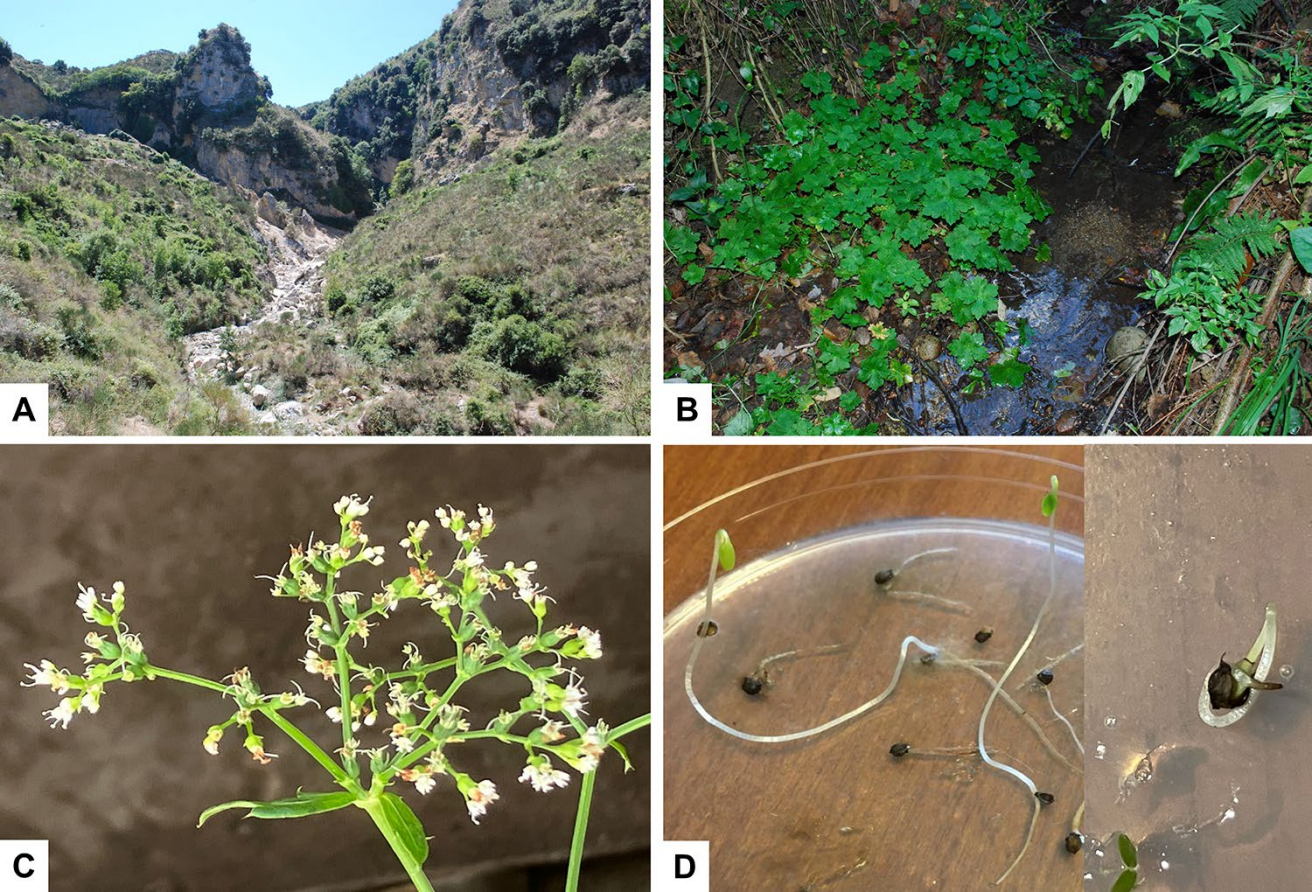
(**A**) Habitat of *Petagnaea gussonei*; (**B**) habit of *P. gussonei*; (**C**) inflorescences of *P. gussonei*; (**D**) germinated seeds of *P. gussonei*.

The plant *Petagnaea gussonei* (Spreng.) Rauschert is an isolated taxon that has affinities with the genera *Sanicula* L. and *Eryngium* L. However, the exact relationships of *P. gussonei* remain unclear^28–31^. Known in Italy as “falsa sanicola” or “Petagna di Gussone” (Fig. 2), it is a perennial herbaceous plant (hemicryptophyte), 20–40 cm high, with peltate basal leaves having five equal lobes and small teeth at the margins; cauline leaves are tripartite and sessile. Inflorescences are in the axil of two opposite tripartite bract leaves. The small, numerous, white flowers are grouped in dichotomy cymes: a central hermaphrodite flower to which three male flowers are attached. According to^31^, each group, with one hermaphrodite flower and three male flowers, should be considered as a highly reduced umbellet, and the inflorescence as a composite umbel. Flowering occurs from early February to May, fruiting from May to July. The fruits are unilocular achenes. Although numerous studies investigated the inflorescence and fruits of *P. gussonei*^28,32,33^, its reproductive biology remains still poorly understood, e.g., with regard to kind of fertilization, pollinators, seeds production and seed viability. *Petagnaea gussonei* can spread rapidly by propagating asexually through rhizome cleavages^34^. This species is restricted to few localities in the Nebrodi Mountains, mostly in inaccessible deep valleys and gorges, within an altitude range of 300–1400 m^35^. The species occurs on hydromorphic soil along small streams and on the edge of rivulets where water flows slowly^35^. *Petagnaea gussonei* lives mainly in mixed woodland areas of deciduous trees (e.g., *Quercus pubescens* Willd., *Quercus cerris* L., rarely *Fagus sylvatica* L.), but it can be also found in hazel groves (*Corylus avellana* L.)^19^.

### Occurrence data of *Petagnaea gussonei*

Localities with the presence of *P. gussonei* were obtained from different sources^19,36,37^, and direct exploration across the Nebrodi Mountains (Table 1). Only records with global positioning system coordinates and detailed localities were used. This study included the occurrences covering the known geographic distribution of the species. Records with uncertain locations, or localities where the species became extinct, were removed. *Petagnaea gussonei* occurs partly within the Nebrodi Park and other smaller nature reserves, partly in various sites not included in protected areas (Fig. 1). During field exploration, an occurrence site never recorded before was discovered in a remote place (site n. 20, see Fig. 1 and Table 1).

**Table 1:** Occurrence sites of *Petagnaea gussonei*.

Site number	Site name	Municipality districts	Coordinates	Altitude (m asl)	Protection status
1	Vallone Mangalaviti	Longi	37° 58′ N–14° 46′ E	1230	Nebrodi Park
2	Stagno Mangalaviti	Longi	37° 57′ N–14° 46′ E	1300	Nebrodi Park
3	Torrente Contrada Contrasto	Longi	37° 57′ N–14° 46′ E	1330	Nebrodi Park
4	Affluente Vallone Linari (Contrada Cufò)	Galati Mamertino	37° 59′ N–14° 46′ E	1150	Nebrodi Park
5	Affluente Vallone S. Pietro (Contrada Cufò)	Galati Mamertino	37° 59′ N–14° 46′ E	1180	Nebrodi Park
6	Vallone Arcangelo (Contrada Pulici)	Galati Mamertino	37° 59′ N–14° 47′ E	1150	Nebrodi Park
7	Vallone Solazzo Salmieri (Contrada Acquasanta)	Tortorici	37° 56′ N–14° 51′ E	1400	Nebrodi Park
8	Vallone Runcillo (Contrada Acquasanta)	Tortorici	37° 56′ N–14° 51′ E	1400	Nebrodi Park
9	Torrente Galati (Gole del Catafurco)	Galati Mamertino	37° 59′ N–14° 48′ E	900	Nebrodi Park
10	Torrente Galati (path Madonna del Catafurco)	Galati Mamertino	37° 59′ N–14° 48′ E	890	Nebrodi Park
11	Torrente Suta (Contrada Monacelle)	Galati Mamertino	38° 00′ N–14° 48′ E	920	Nebrodi Park
12	Vallone Crocetta	Longi	38° 00′ N–14° 45′ E	760	No protected area
13	Sorgente Patirà	Tortorici	38° 01′ N–14° 48′ E	780	Natura 2000 site
14	Vallone Calagna	Tortorici	38° 01′ N–14° 48′ E	600	Nature Reserve
15	Vallone Calagna (reserve entrance)	Tortorici	38° 01′ N–14° 49′ E	520	Nature Reserve
16	Vallone Calagna (west periphery of Tortorici)	Tortorici	38° 01′ N–14° 49′ E	490	Nature Reserve
17	Torrente Fiumetto	Galati Mamertino	38° 01′ N–14° 47′ E	600	Natura 2000 site
18	Torrente Fiumetto (near Pizzo Bufana)	Galati Mamertino	38° 02′ N–14° 47′ E	500	Natura 2000 site
19	Torrente Potame	Tortorici	38° 02′ N–14° 50′ E	700	No protected area
20	Vallone Sant’Andrea	Tortorici	38° 01′ N–14° 51′ E	840	No protected area
21	Torrente Monte Cuculone	Ucria	38° 01′ N–14° 53′ E	850	No protected area
22	Vallone Ruggeri	S. Salvatore di Fitalia	38° 04′ N–14° 47′ E	500	No protected area
23	Vallone Vina	S. Salvatore di Fitalia	38° 04′ N–14° 48′ E	370	No protected area
24	Vallone S. Adriano Vecchio	S. Salvatore di Fitalia	38° 03′ N–14° 48′ E	450	No protected area
25	Vallone S. Lucia	S. Salvatore di Fitalia	38° 03′ N–14° 48′ E	400	No protected area
26	Sorgente Castell’Umberto	Castell’Umberto	38° 05′ N–14° 48′ E	600	No protected area

### Germination tests

Little information is available about the germination patterns of *P. gussonei* in response to various regimes of temperature and light^24^. Numerous studies investigated instead other taxa of the *Saniculoideae* subfamily such as *Sanicula* L.^38,39^, *Astrantia* L.^40^, *Alepidea* F. Delaroche^41^, *Eryngium* L.^42,43^. The germination tests of this study aimed to identify the dormancy-breaking requirements of *P. gussonei* in terms of temperature, light, gibberellic acid (GA_3_) and germination medium (agar, blotting paper). All diaspores (hereafter achenes or fruits) of *P. gussonei* were collected in July 2022 from one of the largest native populations along the “Torrente Fiumetto” site (n. 17, Fig. 1, Table 1), which is about 2.5 km long, with an altitudinal range of 400–750 m asl. Given the generally low fruit production of *P. gussonei* and its endangered conservation status, the “Torrente Fiumetto” population was selected because of its highest density and capacity of reproductive output. Specifically, in agreement with national and international protocols^44,45^, fruits were randomly collected from at least 20 different individuals of *P. gussonei*, each of them far away from the others at a variable distance of 10–50 m, along an altitudinal gradient (400–750 m asl), in order to obtain an adequate degree of genetic diversity. The mean fruit mass was calculated by weighing 5 replicates of 20 fruits. The average weight (for 100 fruits) was measured using a balance with an accuracy of 0.001 g. The dimensions (length, width) of 10 randomly selected fruits were measured using a stereoscopic microscope. Fruit details were 100-fruit mass: 0.27 g ± 0.03; fruit length: 3.6 mm ± 0.32; fruit width: 2.5 mm ± 0.21. According to a revised version of Martin’s^46^ key for types of seeds^47^, *P. gussonei* may have a linear underdeveloped embryo.

All fruits were preliminarily stored in paper bags at controlled conditions (20 °C and 40% of relative humidity), for approximately 3 weeks^44^. Fruits that were not used in the tests were stored at − 18/ − 20 °C for long-term ex situ conservation. Germination tests were performed in the laboratories of the Germplasm Bank of the Department of Biological, Geological and Environmental Sciences (Catania University, Italy). Fruits used in the experiments were washed with sterile distilled water, were not scarified and accompanying structures were not removed. All tests were carried out in temperature and light controlled growth chambers. The light in each chamber was provided by white fluorescent tubes (Osram FL 40 SS W/37), with photosynthetic photon flux density of 40 μ mol m^−2^ s^−1^. To assess the seed dormancy breaking of *P. gussonei*, both 1% agar and blotting paper, combined with 0 (control), 250 mg L^−1^ gibberellic acid (GA_3_) solution, were used at different constant thermoperiods (10 and 15 °C), and at the photoperiod of 12/12 h (light/dark). Each treatment was carried out with four replicates of 20 achenes put in 9-cm Petri dishes. A total of four different experimental treatments were carried out: 1) blotting paper (three layers) moistened with GA_3_ at 10 °C constant, 12/12 h; 2) blotting paper (three layers) moistened with GA_3_ at 15 °C constant, 12/12 h (in treatments 1 and 2, proper GA_3_ concentrations in distilled water were added as needed); 3) agar added with GA_3_ at 10 °C constant, 12/12 h; 4) agar added with GA_3_ at 15 °C constant, 12/12 h.

Optimal germination at lower temperature is a characteristic of Mediterranean plants^48^. Low incubation temperatures were chosen because they seem the most suitable for the germination of *P. gussonei* under natural conditions. Cold temperatures are specifically required to break the dormancy of *P. gussonei*^24^. Low-temperature requirement for dormancy break has been frequently observed in *Saniculoideae* species^39^, resulting in late winter or in early spring germination and emergence of seedlings. Seeds were considered to have germinated when radicle was 1-mm long^49^. All experimental treatments lasted 60 days and were checked every 24 h. At the end of the incubation period, the viability of each remaining fruit was estimated by cut test. Each fruit was classified as viable/fresh, empty or died^45^. Achenes with white and firm embryos were considered viable. The cut test showed that the 80.1 ± 7.1% seeds are empty (embryoless seeds are quite common in the *Apiaceae*); 15 ± 1.7% are dead; 4.9 ± 0.53% viable (not germinated). Viable seeds that did not germinate at the end of each test were not further investigated.

### Germination parameters

The germination process of *P. gussonei* seeds was analysed through several parameters such as capacity, rate, time, speed and homogeneity, which were calculated with the following formulas:

- *Final germination percentage* (FGP)^50^,
  $${\text{FGP}} = \left( {\frac{{\mathop \sum \nolimits_{i = 1}^{k} n_{i} }}{N}} \right) \times 100$$
  where *n*_*i*_ = number of seeds germinated on the *ith* day; *k* = 60, number of days of experiment duration; *N* = 20, total number of seeds put in a Petri dish;
- *Mean germination time* (MGT)^51^,
  $$MGT = \frac{{\mathop \sum \nolimits_{i = 1}^{k} n_{i} t_{i} }}{{\mathop \sum \nolimits_{i = 1}^{K} n_{i} }}$$
  where *t*_*i*_ = number of days between the start of the experiment and the *ith* day;
- *Mean germination rate* (MGR), calculated as the reciprocal of MGR^52^,
  $${\text{MGR}} = 1/{\text{MGT}}$$
- *First day of germination* (FDG), expressed as the day on which the first germination event occurs^53^;
- * Last day of germination* (LDG), expressed as the day on which the last germination event occurs^53^;
- *Median germination time* (T_50_)^54^,
  $$T_{50} = t_{i} + \frac{{\left( {\frac{N + 1}{2} - n_{i} } \right) \times \left( {t_{j} - t_{i} } \right)}}{{n_{j} - n_{i} }}$$
  where *N* = final number of germinated seeds; *n*_*i*_ and *n*_*j*_ are the total number of seeds germinated by adjacent counts at time *t*_*i*_ and *t*_*j*_ , with *n*_*i*_ < *N/2* < *n*_*j*_;
- *Coefficient of variation of the mean germination time* (CV_t_)^52^,
  $${\text{CV}}_{t} = \left( {s_{t} /{\text{MGT}}} \right) \times {1}00$$
  where the variance of the mean germination time is expressed as:
  $$s_{t}^{2} = \frac{{\mathop \sum \nolimits_{i = 1}^{k} n_{i} \left( {t_{i} - {\text{MGT}}} \right)^{2} }}{{\mathop \sum \nolimits_{i = 1}^{k} n_{i} - 1}}$$
- *Coefficient of velocity of germination* (CVG)^55^,
  $${\text{CVG}} = \frac{{\mathop \sum \nolimits_{i = 1}^{k} n_{i} }}{{\mathop \sum \nolimits_{i = 1}^{k} n_{i} t_{i} }} \times 100$$
- *Germination Rate Index* (GR)^56^,
  $${\text{GRI}} = G_{1} /1 + G_{2} /2 + \cdots + G_{i} /i$$
  where *G*_1_ is the final germination percentage (*FGP*_1_) on day 1, *G*_2_ is the final germination percentage (*FGP*_2_) on day 2, and so on;
- *Germination Index* (GI)^57^,
  $${\text{GI }} = \left( {{6}0 \, \times {\text{ n}}_{{1}} } \right) + \left( {{59 } \times {\text{ n}}_{{2}} } \right) + \cdots + \left( {{1 } \times {\text{ n}}_{{{6}0}} } \right)$$
  where n_1_, n_2_,…, n_60_, are respectively the number of seeds germinated on the first, second,…*ith* day.

### Climate trends: multi-temporal processing

**Figure 3:**
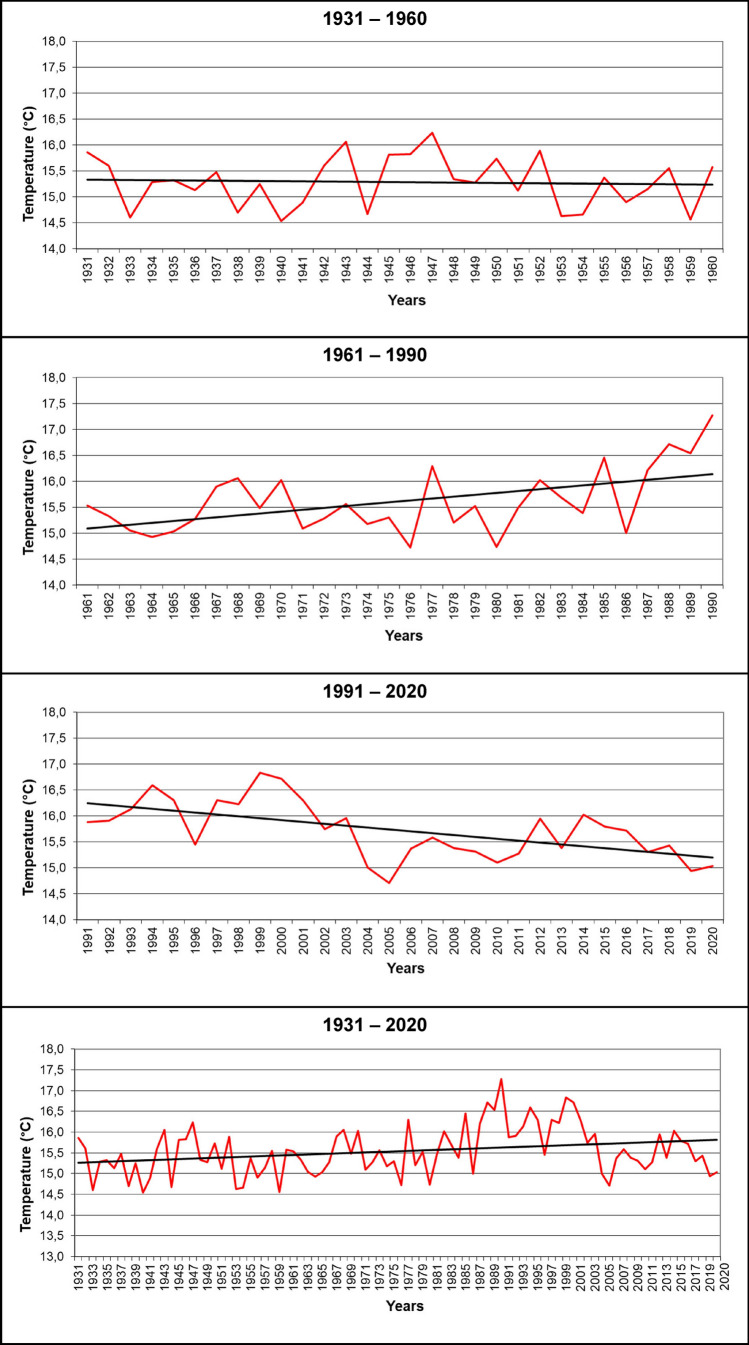
Temperature trends in the distributional area of *Petagnaea gussonei* over 1931–1960, 1961–1990, 1991–2020, 1931–2020.

**Figure 4:**
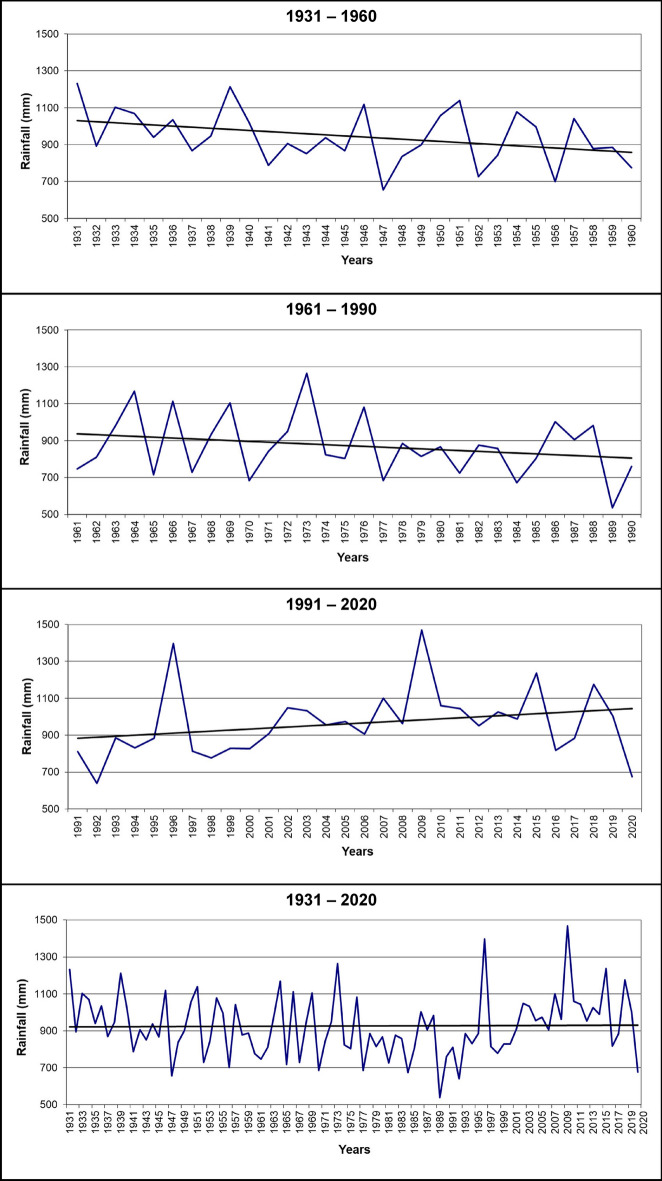
Rainfall trends in the distributional area of *Petagnaea gussonei* over 1931–1960, 1961–1990, 1991–2020, 1931–2020.

This study aimed to identify the trends of annual temperature and rainfall in the distributional area of *P. gussonei* (Figs. 3 and 4). Specifically, the variations of temperature and rainfall were processed over a 90-year period, distributed in four temporal intervals: 1931–1960, 1961–1990, 1991–2020, 1931–2020. The raw data were recorded by the weather stations within the natural range of *P. gussonei*, and were acquired from the website of the Sicilian Regional Government^58^. The annual mean values of temperature and rainfall, and their resulting trends, were processed and arranged in an excel spreadsheet.

### Areas sensitive to desertification: multi-temporal processing

**Figure 5:**
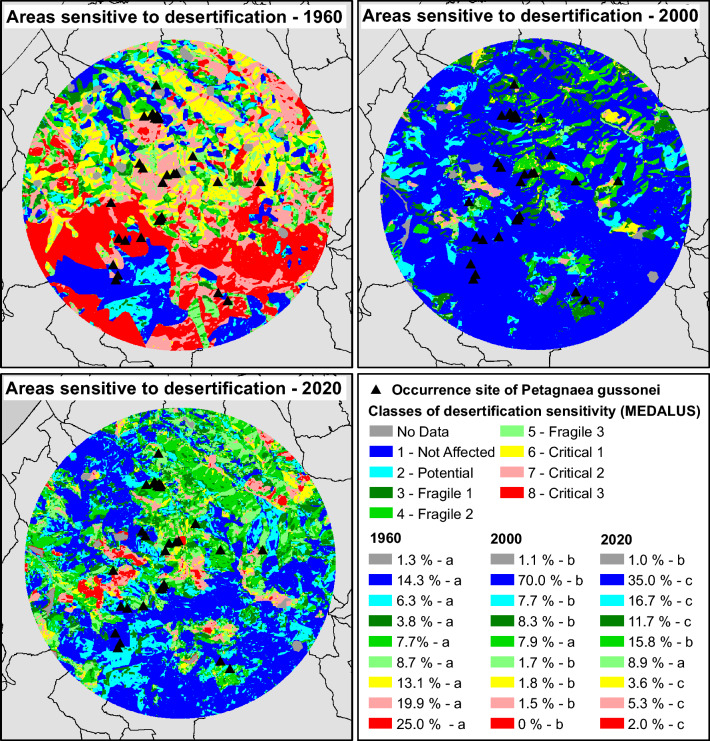
Areas sensitive to desertification in 1960, 2000, 2020, within the natural range of *Petagnaea gussonei* (different letters show significant differences – *p* < 0.05 – among 1960, 2000 and 2020 within the same class).

Desertification trend in the distributional area of *P. gussonei* was processed through the MEDALUS approach (Mediterranean Desertification and Land Use), funded by the European Commission^59^. The desertification sensitive areas are identified on the basis of an index (Environmental Sensitive Areas to Desertification index, ESAI) that integrates bio-physical data on environmental quality (climate, soil and vegetation) with management (anthropogenic) factors^60^. Three types of environmental sensitive areas (ESAs) to desertification can be distinguished in the MEDALUS approach (Fig. 5): (a) critical ESAs for areas already highly degraded, (b) fragile ESAs for areas in which any change in the delicate natural and human activity balance can lead to desertification, and (c) potential ESAs for areas threatened under significant climate change or if a particular combination of land use practices is implemented. The software ArcGIS version 10.2 was used to process ESAs.

### CORINE Land Cover: multi-temporal processing

**Figure 6:**
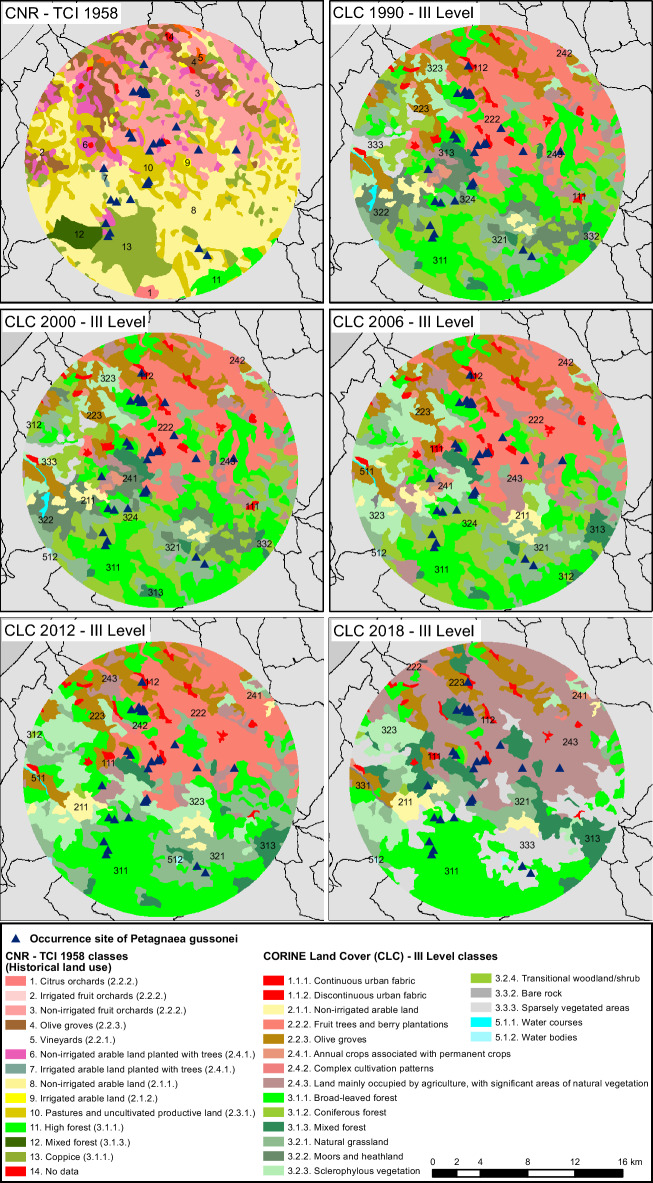
Soil-use changes according to CNR-TCI in 1958, and CORINE Land Cover (CLC) III level in 1990, 2000, 2006, 2012, 2018, within the natural range of *Petagnaea gussonei*.

The European CORINE Land Cover (CLC) project was used to investigate the soil-use changes occurred in the natural range of *P. gussonei*. In particular, CLC III level was used to identify soil-use classes in 1990, 2000, 2006, 2012 and 2018 (Fig. 6). The CLC GIS data were collected from SINANET^61^. Regarding soil uses in 1958, this study obtained land cover data from the “map of Italy’s soil-use”^62^. The equivalence between CNR-TCI classes and CLC classes was reported in Fig. 6. All soil-use data were turned into shapefiles, which were then processed through the software ArcGIS version 10.2.

### Statistical analysis

The Wilcoxon signed ranks test was used when two related samples were compared. In case of unrelated pairs of samples, the Mann–Whitney *U*-test was carried out. The Friedman test was instead used when the comparison of multiple related samples was needed. The Kruskal–Wallis *H*-test was carried out when multiple unrelated samples were compared. To detect significant differences between pairs, contrasts were carried out with the Wilcoxon signed ranks test (related pairs) and the Mann–Whitney *U*-test (unrelated pairs). Conducting multiple contrasts may increase the Type I error rate. The initial level of risk (α = 0.05) was therefore adapted according to the Bonferroni formula α_B_ = α/k, where α_B_ is the adapted level of risk, and k is the number of comparisons. The Kendall rank correlation coefficient was applied to check for significant monotonic correlations. The degree of significance was set at 0.05. Statistical processing was performed with the statistical package IBM SPSS Version 27.0.

### Plant materials statement

The plant collection and use were in accordance with all the relevant guidelines. Collection permission was granted by the Italian Ministry of the Environment. Specimens were collected and identified by the authors of this article. Voucher specimens were stored at the Seed Bank of the Department of Biological, Geological and Environmental Sciences (Catania University, Italy).

## Results and discussion

### Germination behavior of *Petagnaea gussonei*

The final germination percentage (FGP) of the four experimental treatments showed significantly different values, which ranged from 14% (GA_3_, 15 °C, no agar) to 32% (GA_3_, 10 °C, with agar) (Table 2). The results suggest that the germination behavior of *P. gussonei* is not only sensitive to modest temperature oscillations (10–15 °C), but also to the presence of agar as germination medium. Indeed, at 10 °C, FGP varied from 18% (no agar) to 32% (with agar); similarly, at 15 °C, FGP was 14% without agar, and 23% with agar. In particular, germination capacity benefited from lower temperatures by shifting from 14% (15 °C) to 18% (10 °C), and the use of agar as growth medium acted as a stronger germination booster thanks to which FGP increased by 14% at 10 °C, and by 9.0% at 15 °C. No achene germinated under the control condition during the whole experimental period. The best result of FGP was 32%, which was obtained at 10 °C and with agar, thus implying that low temperatures (10 °C) and grow medium (agar) are the best experimental germination conditions.

**Table 2:** Germination parameters of *Petagnaea gussonei*.

Treatment	FGP (%)	MGT (days)	MGR	FDG (days)	LDG (days)	CV_t_	CVG	GRI (%/day)	GI	T_50_ (days)
GA_3_, blotting paper, 10 °C	17 ± 2.4^a^	30.3 ± 2.4^a^	0.033 ± 0.004^a^	26 ± 3.1^a^	39 ± 5.2^a^	14.9 ± 2.3^a^	3.3 ± 0.41^a^	0.50 ± 0.07^a^	92 ± 12.3^a^	28.3 ± 2.9^a^
GA_3_, blotting paper, 15 °C	14 ± 1.6^b^	36 ± 4.1^b^	0.028 ± 0.003^b^	32 ± 2.7^b^	40 ± 3.5^b^	15.7 ± 1.9^b^	2.8 ± 0.31^b^	0.28 ± 0.03^b^	50 ± 4.3^b^	32 ± 2.6^b^
GA_3_, agar, 10 °C	32 ± 2.5^c^	32.5 ± 3.5^c^	0.031 ± 0.02^c^	25 ± 2.1^c^	37 ± 3.0^c^	15.8 ± 1.4^c^	3.1 ± 0.23^c^	0.94 ± 0.07^c^	171 ± 13.2^c^	32 ± 2.4^c^
GA_3_, agar, 15 °C	23 ± 1.9^d^	34.4 ± 2.9^d^	0.029 ± 0.002^d^	29 ± 2.2^d^	42 ± 3.3^d^	10.4 ± 1.2^d^	2.9 ± 0.23^d^	0.73 ± 0.06^d^	133 ± 10.6^d^	33.5 ± 2.8^d^

1) FGP, final germination percentage; MGT, mean germination time; MGR, mean germination rate; FDG, first day of germination; LDG, last day of germination; CV_t_, coefficient of variation of germination time; CVG, coefficient of velocity of germination; GRI, germination rate index; GI, germination index; T_50_, median germination time; 2) different letters show significantly different values, among different treatments, within the same parameter (*p* < 0.05).

The values of the mean germination time (MGT) showed that the variation of temperature played instead a primary role in influencing germination velocity (Table 2). Specifically, at 10 °C, MGT values were 30.3 days (no agar) and 32.5 days (with agar), whereas at 15 °C, the values were 36 days (no agar) and 34.4 (with agar). The seeds of *P. gussonei* reported thus a faster germination at lower temperatures (10 °C), in agreement with the first day of germination (FDG) that was 26 without agar, and 25 with agar. The lower temperatures seem also to prompt the germination of more seeds on the same day, as reported by the coefficient of velocity of germination (CVG), whose values at 10 °C were 3.30 (no agar) and 3.08 (with agar), whereas 2.78 (no agar) and 2.91 (with agar) at 15 °C (Table 2). Regarding the germination rate index (GRI) and the germination index (GI), the highest values were found at the lowest temperature (10 °C), specifically (without agar) GRI = 0.50%/day, and GI = 92, which respectively increased to GRI = 0.94%/day, and GI = 171, thanks to the addition of agar. Similarly, the median germination time (T_50_) showed that the fastest results of 50% of the germinated seeds were reached at lower temperature (10 °C) and with agar, in particular T_50_ = 28.3 days (10 °C, no agar), and T_50_ = 31 days (10 °C, with agar). Overall, the germination parameters corroborated the role of low temperatures and agar, whose combined use makes the germination of *P. gussonei* seeds faster and higher.

Studies on dormancy and germination of species of the *Saniculoideae* subfamily are scarce^9^. Only a few attempts have been made to resolve dormancy-breaking requirements in *Saniculoideae* species. However, most studies agree with the general high dormancy in *Saniculoideae* species, including *P. gussonei*^24^ showed that only high concentrations of GA_3_ had some effect on *P. gussonei* seed germination (GA_3_ = 1000 mg l^−1^), which occurred between 4 and 5 weeks in 12 ± 1.2% of the seeds incubated in Petri dishes, in a growth chamber set at 18–23 °C. In particular^24^, pointed out that high temperatures may result in a low germination of *P. gussonei* seeds, whereas low temperatures are an important factor in breaking seed dormancy in *P. gussonei*, as found by these authors for the pots placed in natural conditions, which were exposed to colder temperatures. In another study^38^, investigated the factors affecting dormancy break of *Sanicula europaea* L., which is a species very close to *P. gussonei*, and the only representative of *Sanicula* occurring in western Europe. These authors found that the incubated seeds of *S. europaea*, previously pre-treated with cold stratification, may reach a germination rate of c. 70% in a faster way and at low temperatures of 5–10 °C. Other studies with similar species were in line with the above findings, as found for instance in *Sanicula canadensis* L., *Eryngium maritimum* L. and *Eryngium vuccifolium* Michx., which all require cold temperatures for dormancy break^63–65^. In agreement with dormancy patterns reported for similar species, the seed germination in *P. gussonei* seems significantly favored under low temperatures (5–10 °C). However, *P. gussonei* shows a lower germination rate when compared to other *Saniculoideae* species^38,39,42^.

Most *Apiaceae* species generally have seeds containing an underdeveloped embryo at the moment of dispersal^46^. Prior to germination, the underdeveloped embryo has to grow to a critical length inside the seed. These seeds are defined as morphologically dormant (MD). In seeds of numerous temperate species with MD, an additional physiological obstacle preventing embryo growth and/or germination occurs. Ref^66^ defined this combined dormancy as morpho-physiological dormancy (MPD). Physiological dormancy in MPD seeds is usually broken before or during elongation of the embryo^9^. Therefore, in order to germinate, MPD seeds require a dormancy-breaking pretreatment. The significant role of GA_3_ on breaking *P. gussonei* dormancy may suggest that the seeds of *P. gussonei* seem to have mainly MPD. Moreover, this study showed that *P. gussonei* seeds start germinating after 4–5 weeks, and in MPD seeds, embryo growth/radicle emergence requires a considerably longer period of time than in MD seeds^67^. Similarly, various studies showed that several species of the *Sanicula* genus, closely related to *P. gussonei*, have MPD seeds^38,63,68^. High temperatures and dormancy are undoubtedly two important factors that limit *P. gussonei* germination, which is further affected by the ongoing climate changes.

### Climate trends in the distributional area of *Petagnaea gussonei*

The climate patterns showed fluctuating trends, especially over 30-year intervals (Figs. 3 and 4). In the period 1931–1960, the average values of temperature decreased moderately by 0.2 °C, specifically from 15.4 to 15.2 °C; in the following period of 1961–1990, the average temperature increased very significantly by 1.1 °C, from 15.1 to 16.2 °C; in turn, the last thirty years, 1991–2020, showed a decrease by 1.0 °C, from 16.2 to 15.2 °C. Overall, in the period of 1931–2020, the average temperature increased by 0.5 °C, in particular from 15.4 to 15.9 °C. Similarly, the average values of rainfall showed oscillations over periods of thirty years (Fig. 4): during 1931–1960, the average rainfall declined by 200 mm, from 1050 to 850 mm; in the time interval of 1961–1990, the decrease in rainfall from 950 to 800 mm, thus by 150 mm, was significant but slightly lower; instead, in the final period of 1991–2020, the average rainfall rose by 170 mm, namely from 880 to 1050 mm. However, in the global period of 1931–2020, although the general trend of the average rainfall showed a relatively constant value of 920 mm, the significant decline in rainfall during the first 60 years (1931–1960 and 1961–1990) was overall compensated by the increase in rainfall in the last 30 years (1991–2020), during which the rainfall regime of the study area showed considerable extreme events.

The Mediterranean region is warming and becoming increasing arid^69^. Rainfall has begun to either decrease in the long term, mainly in the dry season, or, as found in this study, not to change significantly^70^. In either case, however, a steady increase in temperatures has led to greater aridity, which generates frequent extreme events such as floods, severe heat, longer droughts and wildfires^71–73^. Moreover, this study showed that the rise by 0.5 °C during 1931–2020 is overall in line with future climate change projections, which expect an increase in air temperatures by 0.3–4.0 °C throughout the twenty-first century over the Mediterranean region, under an intermediate emission climate change scenario^69^. Given the high evidence of large climate shifts, the Mediterranean region has been identified as a hotspot of future climate changes^74–76^. This ongoing climate crisis is inevitably worsening the conditions of natural ecosystems and wildlife in the Mediterranean region, which is considered as a global biodiversity hotspot^6,77,78^. The Mediterranean biodiversity, indeed, accounts for c. 20% of world’s vascular plants^79^. In particular, the combination of a marked seasonality, long-term fluctuations, and a diversified geological mosaic, favored an exceptional accumulation of endemic plants^80^, among which 36% are narrow endemics, i.e., they grow only in a single area or have a narrow geographic range^81^. Many of these endemic species persisted over geological times^82,83^, in areas where the local (or regional) climate was buffered in comparison with the global climate changes. These areas are named refugia^84^, and many of them are located in the mountains of southern Europe^85^, where heterogenous topography results in a variety of microclimates providing suitable habitats during both warm and cold periods^86–88^. In the long term, these refugia not only provided suitable habitats for many taxa from the Northern Europe, but also acted as the locations of post-glacial recolonization when temperatures rose again at the end of the Ice Age^89–91^.

The species of this study, namely *P. gussonei*, shows the above mentioned biological, geological and geographical peculiarities of Mediterranean plant biodiversity. *Petagnaea gussonei* is indeed a typical endemic plant distributed within a very narrow area (< 20-km diameter) acting as refugia across a southern Mediterranean mountain range (Nebrodi Mountains), which is also geologically old, complex and heterogenous^25,26^. However, current predictions of climate changes show serious threats to this unique biological heritage. Even minor atmospheric shifts can lead to substantial changes in climate, making Mediterranean mountains potentially more vulnerable than the other Mediterranean zones^92–94^. Some predictions report that a change in the biogeographic characteristics of these mountains is already occurring^95–97^. With increasing temperatures, many species across the world have already shifted their ranges to more suitable habitats, moving upwards in elevation or towards the poles^98,99^, especially in mountains and at high elevation^100^. Moreover, although many plant endemics are protected in reserves, some species may locally disappear because their niche lower limit passes over the local highest elevation^101^. In particular, current reserve networks, in the absence of functional corridors, may be inadequate on any scale to ensure long-term persistence of rare and endangered species^102^. It is generally believed that the risk of extinction under climate changes strongly reflects the inability of species to shift to suitable habitats^103^. However, the velocity of the predicted climate changes is very likely to exceed the migration capability of many range-restricted species^104^, especially because these species are often habitat specialists and weak dispersers. This is the case of Mediterranean narrow mountain endemics, like *P. gussonei*, which are expected to be particularly sensitive to environmental shifts, as several of these plants are extremely specialized and have evolved low dispersal ability^105–107^. Climate changes alter also the main stages of plant phenology^108^ and sexual reproduction^109^, thus making these species more vulnerable to meteorological extreme events, and affecting plant–pollinator interaction^110^. However, still few studies have examined the effects of future climate changes on Mediterranean plant species, mainly on woody species, whereas information on endemic or rare herbaceous plants is even poorer. It is therefore of the utmost importance to assess the impact of climate changes on Mediterranean plant biodiversity, with a special focus on range-restricted mountain endemics like *P. gussonei*.

### Desertification trends in the distributional area of *Petagnaea gussonei*

The areas sensitive to desertification showed overall different trends across the tree study periods (Fig. 5). Firstly, it is considerable how the “not affected” areas (class 1) increased from 14.3% in 1960, to 70.0% in 2000. During this 40-year period (1960–2000), the significant expansion of areas not sensitive to desertification is likely to reflect the positive events that occurred within the distributional range of *P. gussonei*. These events include the abandoning of cultivated fields, the consequent recovery and spread of natural vegetation, as well as the creation of wide protected areas such as the Nebrodi Mountains Park, which is the largest nature reserve in Sicily covering a surface of c. 86,000 ha. In the period 1960–2000 (Fig. 5), the results of this study showed also a moderate-low increase in the areas classified as potential, fragile 1 and fragile 2; in turn, the categories fragile 3, critical 1, critical 2, critical 3 reported a significant decline, the most considerable of which was the category “critical 3” that decreased from 25 to 0%. However, the general positive trend of desertification during 1960–2000 suffered a significant reversal of the tendency in the period 2000–2020 (Fig. 5). During this 20-year interval, all classes showed significantly worse values in the distributional area of *P. gussonei*: the increase in areas sensitive to desertification was on average two and tree fold compared to the previous 40-year period. In particular, the most dramatic result was the collapse of the “not affected” areas, which declined by half from 70% (2000) to 35% (2020). The factors, which contributed to this serious worsening of the areas sensitive to desertification, likely include high human impact with consequent natural habitat alteration, and massive erosive phenomena associated with extreme events, such as violent rains resulting from the ongoing climate changes.

Soil and landscape degradation, driven by human activities and climate variation, are considered key factors of desertification^111^. Degraded soils, in particular, often show significant erosion processes that can be due to, e.g., unsustainable land management^112^. Inappropriate changes in soil use include, on the one hand, removal or disturbance of surface cover, e.g., by fire, cultivation, intensive tillage and ploughing^113,114^; on the other hand, loss of vegetation cover and lack of adequate soil conservation practices^112^. Climate changes resulting in more intense storms may also be an important driver of soil erosion^115^. However, desertification occurs at different temporal and spatial scales, and is influenced by several factors that make the assessment of the process extremely complex^116^. Moreover, robust information is often insufficient to provide indicators that allow precise and efficient measurement of desertification effects^117^. The distributional range of *P. gussonei* falls within the heart of a hotspot of desertification: the Mediterranean basin. The Mediterranean region has been identified as particularly vulnerable to soil degradation^14^. In 2017, 25% of European land (411,000 km^2^), especially in southern Europe, was identified as being at high or very high risk of desertification, a 14% increase since 2008^118^. The Mediterranean region has the overall highest erosion rates within the EU^119^, the lowest levels of soil organic matter^120^, and severe salinization problems^121^. It also has high abundance of shallow soils^122^, strong and increasing human pressures^123^, and high climate change vulnerability^13,16^. The main causes of soil degradation and increasing land sensitivity to desertification in the Mediterranean basin are primarily human-induced^124^, and are generally more pronounced in areas with semi-arid or dry climate conditions, with water being the main factor limiting ecosystem performance, resilience and recovery^125,126^. In rural areas, soil degradation occurs mainly through deforestation or unsustainable cropping, irrigation or grazing practices, which, in turn, stem from the socioeconomic conditions of the local people^127^.

Across the Mediterranean region, increasing abandonment of extensive livestock systems raises wildfire risk and affects the current land footprint through changes in the mosaic landscape^128^. Changes to agricultural systems, together with other land-use changes, are leading to critical levels of habitat loss^16^. This is of particular concern because the Mediterranean region is characterized by extraordinary biodiversity, with large numbers of endemic species^129^. Unfortunately, the endemic plant *P. gussonei* is distributed in an area prone to land degradation and desertification due to both unfavorable bio-physical conditions and adverse human activities. Such factors include erratic precipitation (occurring mainly in winter season), high summer temperature and frequent seasonal droughts, poor and erodible soils, diversified landscapes, extensive human-induced deforestation and forest losses due to frequent wildfires, land abandonment and deterioration. Managing land in a sustainable manner means using land without damaging ecological processes or reducing biological diversity. It requires the maintenance of key components of the environment, such as biodiversity, ecological integrity and natural capital, as well as the conservation of a rich set of relationships among the constitutive elements of the system. Sustainable land management is a straightforward tool not only for the recovery of arid land, but also for the conservation of local biodiversity hotspots such as the Nebrodi Mountains where *P. gussonei* grows, and where landscape resilience could be improved by creating further protected areas and by increasing vegetation cover.

### Soil-use changes in the distributional area of *Petagnaea gussonei*

CORINE Land Cover (CLC) classes showed numerous and complex changes from 1958 to 2018 (Table 3). Continuous and discontinuous urban fabric occupies just c. 1.5% of the *P. gussonei* range, and was constant across the 60-year study period. Agricultural areas (CLC class 2) declined considerably from 83.6% in 1958 to 40.3% in 2018. Forests and semi-natural areas (CLC class 3), instead, increased significantly from 16% in 1958 to 58.1% in 2018. Water bodies (CLC class 5) showed very low values, which were c. 0.3% in 1958 and less than 0.1% in 2018. Regarding the agricultural areas, the dominant class in 2018 was land mainly occupied by agriculture, with significant areas of natural vegetation (2.4.3.); fruit trees and berry plantations (class 2.2.2.) were dominant from 1990 to 2012 (20.4–22.2%), but drastically declined to 0.4% in 2018. Among forests and semi-natural areas, the dominant class throughout the study period was broad-leaved forest (3.1.1.), which reached a peak of 28.1% in 2012, and moderately declined to 22% in 2018. Other significant classes were sparsely vegetated areas (3.3.3.) and mixed forest (3.1.3.), which in 2018 were respectively 13.6 and 9.8% of the distributional area of *P. gussonei*. Overall, the occurrence sites of *P. gussonei* benefited from a drastic decrease in agricultural areas, and from a significant increase in forested and semi-natural areas, especially in terms of broad-leaved and mixed forests. However, farmland still occupies 40.3% of *P. gussonei* natural range, and 13.6% of the study area is characterized by sparse vegetation covering only 10–50% of surface, which is therefore highly vulnerable to erosion processes.

**Table 3:** Areas (ha) of soil-use classes in 1958, 1990, 2000, 2006, 2012, 2018, within the natural range of *Petagnaea gussonei*.

CORINE Land Cover classes	1958*		1990		2000		2006		2012		2018	
	ha	%	ha	%	ha	%	ha	%	ha	%	ha	%
1.1.1. Continuous urban fabric	n.a	n.a	230^a^	0.5	239^a^	0.6	194^b^	0.5	203^c^	0.5	205^c^	0.5
1.1.2. Discontinuous urban fabric	n.a	n.a	424^a^	1.0	448^a^	1.1	448^a^	1.1	436^a^	1.0	436^a^	1.0
2.1.1. Non-irrigated arable land	13,637^a^	32.1	658^b^	1.6	658^b^	1.6	954^c^	2.3	1153^d^	2.7	1111^d^	2.6
2.1.2. Irrigated arable land	179	0.4	0	0.0	0	0.0	0	0.0	0	0.0	0	0.0
2.2.1. Vineyards	269	0.6	0	0.0	0	0.0	0	0.0	0	0.0	0	0.0
2.2.2. Fruit trees and berry plantations	9304^a^	21.9	8658^b^	20.4	8646^b^	20.4	9387^c^	22.2	9484^c^	22.4	150^d^	0.4
2.2.3. Olive groves	2962^a^	7.0	3991^b^	9.4	3970^b^	9.4	3970^b^	9.4	3870^c^	9.1	2809^d^	6.6
2.3.1. Pastures and uncultivated productive land	6804	16.0	0	0.0	0	0.0	0	0.0	0	0.0	0	0.0
2.4.1. Annual crops associated with permanent crops	2362^a^	5.6	192^b^	0.5	192^b^	0.5	192^b^	0.5	80^c^	0.2	119^d^	0.3
2.4.2. Complex cultivation patterns	n.a	n.a	134^a^	0.3	134^a^	0.3	134^a^	0.3	110^b^	0.3	68^c^	0.2
2.4.3. Land mainly occupied by agriculture, with significant areas of natural vegetation	n.a	n.a	1623^a^	3.8	1623^a^	3.8	3720^b^	8.8	1932^c^	4.6	12,792^d^	30.2
3.1.1. Broad-leaved forest	5948^a^	14.1	9125^b^	21.5	9125^b^	21.5	7124^c^	16.8	11,912^d^	28.1	9316^e^	22.0
3.1.2. Coniferous forest	n.a	n.a	74^a^	0.2	74^a^	0.2	130^b^	0.3	72^a^	0.2	72^a^	0.2
3.1.3. Mixed forest	798^a^	1.9	784^a^	1.9	784^a^	1.9	1263^b^	3.0	1506^c^	3.6	4149^d^	9.8
3.2.1. Natural grassland	n.a	n.a	4264^a^	10.1	4264^a^	10.1	4907^b^	11.6	6440^c^	15.2	2575^d^	6.1
3.2.2. Moors and heathland	n.a	n.a	3118^a^	7.4	3118^a^	7.4	n.a	n.a	n.a	n.a	2,642^b^	6.2
3.2.3. Sclerophylous vegetation	n.a	n.a	1545^a^	3.6	1545^a^	3.6	4501^b^	10.6	5018^c^	11.8	51^d^	0.1
3.2.4. Transitional woodland/shrub	n.a	n.a	6630^a^	15.6	6630^a^	15.6	5,352^b^	12.6	75^c^	0.2	61^d^	0.1
3.3.2. Bare rock	n.a	n.a	86^a^	0.2	86^a^	0.2	n.a	n.a	n.a	n.a	n.a	n.a
3.3.3. Sparsely vegetated areas	n.a	n.a	710^a^	1.7	710^a^	1.7	43^b^	0.1	n.a	n.a	5766^c^	13.6
5.1.1. Water courses	n.a	n.a	112^a^	0.3	112^a^	0.3	38^b^	0.1	41^c^	0.1	n.a	n.a
5.1.2. Water bodies	n.a	n.a	9^a^	0.02	9^a^	0.02	9^a^	0.02	36^b^	0.09	36^b^	0.09

“n.a.” is data not available; *soil-use classes in 1958 are provided by CNR-TCI, whose equivalence with CORINE Land Cover classes is reported in Fig. 6; different letters show significantly different values, among different years, within the same CORINE Land Cover class.

Landscape transformation is a key threat to ecosystem integrity, and the negative impacts extend far beyond the boundaries of the transformed land^130,131^. Land-use changes caused by human activities have contributed directly to global biodiversity loss^132^. Land-use changes, in particular, are considered the major cause of natural habitat fragmentation and alteration due to the sprawl of rural and urban areas^133^. Habitat fragmentation may decrease species diversity directly or indirectly by reducing the core area or causing habitat isolation^134,135^. Fragmentation reduces also habitat connectivity and may increase the risk of extinction by altering the spatial configuration and community composition^17^. Halting biodiversity loss attributed to anthropogenic drivers, such as land-use changes, is thus one of the most urgent challenges for human society. For terrestrial ecosystems, the conversion of natural habitats to agricultural land is the primary threat^136^. An estimated 40% of the Earth’s ice-free land has already been converted to cropland and pastures^137,138^. This global estimate is in line with the study area whose agricultural component decreased by half over a 60-year period. Despite this seemingly positive trend, the natural range of *P. gussonei* still falls within an area significantly impacted by farmland. However, the dominant CORINE agricultural class of the study area is defined as “*land mainly occupied by agriculture*” but also “*with significant areas of natural vegetation*” (2.4.3.), thus suggesting that landscape connectivity can be a feasible and effective guiding principle of conservation. To prevent local species extinctions, and to ensure the long-term maintenance of biodiversity, it is necessary to connect patches of remaining natural habitat^139^. In this context, the implementation of conservation networks of natural or restored vegetation have become a favored mitigation approach in regions where arable cropping occurs within naturally forested landscapes^140,141^. These ecological networks comprise mostly linear patches of remnant land (often connecting protected areas outside ecological networks), connected together through corridors and stepping-stone habitats^142,143^.

Ecological networks are an effective approach that integrate environmental management strategies and landscape planning, and can be understood by different actors^144–146^. However, landscape connectivity for plants is mainly linked to their ability to disperse between habitat patches via propagules. Their dispersal is only successful if habitat patches are sufficiently connected^147,148^, or if it is facilitated by suitable landscape features^149^. Indeed, the ability of plants to disperse in fragmented landscapes also depends on their dispersal strategy^150,151^. Overall, biodiversity loss largely depends on the temporal patterns of environmental changes, e.g., when biological response cannot match the rate of soil-use changes^152^. Whatever their alteration, restoring landscapes to their primitive ecological configuration is conservation utopia. Reference environmental conditions cannot exist because Earth’s systems change in a natural way. Moreover, landscapes with a mosaic of human-affected soil uses may be biodiversity-friendly only to a certain point, but not completely. Improving landscape connectivity should be considered as a flexible and proactive solution for preserving biodiversity in most areas with different patterns of land-use changes, including the distributional area of *P. gussonei*, which can greatly benefit from dispersal corridors, especially because of its high capacity of asexual reproduction. Dispersal corridors provide also habitats for pollinators, which may contribute to the relatively low sexual reproduction of *P. gussonei*. Besides global and regional drivers of biodiversity decline, the consequences of local land-use decisions are major factors of this global decline, but they can be also the starting point for a biodiversity recovery.

## Conclusions

The endemic plant *P. gussonei* showed critical conditions due to exogenous and endogenous factors. Dormancy affects endogenously *P. gussonei*, whose poor germination capacity may be further lower and slower as a result of the ongoing climate changes. Species like *P. gussonei*, with peculiar requirements for breaking dormancy, make inevitably the role of germplasm banks fundamental in ex situ conservation programs. Species respond also to exogenous factors that may act on different space–time scales. The natural range of *P. gussonei* falls within a narrow area where the exogenous impact of climate changes and desertification showed alarming trends, which especially worsened in the last few decades. Similarly, *P. gussonei* is distributed across a complex mosaic where the exogenous action of soil-use changes created a fragmented landscape. This conservation issue seems a dead end, but a feasible approach is possible to tackle it. The first step is to consider that these exogenous and endogenous factors interact, therefore coping with one factor helps dealing with the other three. Improving ecological connectivity can be a starting point to reverse the trends of the three exogenous factors, and to reduce the endogenous factor by favoring the dormancy-breaking of *P. gussonei*. Indeed, creating ecological networks is a realistic approach to conservation because c. 30% of *P. gussonei* range is characterized by “*land mainly occupied by agriculture*” but “*with significant areas of natural vegetation*”, which can be interconnected through dispersal corridors. The genic flux of the *P. gussonei* metapopulation will benefit from widespread landscape connectivity, thus compensating the limiting consequences of a high dormancy. However, other biodiversity-friendly interventions should be considered, such as increasing plant cover in those areas with scarse vegetation in order to mitigate the erosion processes associated with desertification. All these actions will contribute to make *P. gussonei* more resilient to the ongoing climate changes. The second step is political, and consists in involving different stakeholders according to the kind of factor. Scientists have to continue their job to shed further light on the ecophysiology of *P. gussonei* seeds. Local communities play an invaluable role in influencing the soil uses that shape the distributional area of *P. gussonei*. Policy makers act on a wider scale, and can take concrete actions against climate changes by setting their agenda. A better biodiversity-friendly world is possible only if each stakeholder does its part and is open to dialogue with the other stakeholders.

## Data Availability

The data that support the findings of this study are not openly available due to reasons of sensitivity, and are available from the corresponding author upon reasonable request. Data are located in controlled access data storage at the Department of Biological, Geological and Environmental Sciences (Catania University, Italy).
